# The Dyad-Adaptive Paced Auditory Serial Addition Test (DA-PASAT): Normative data and the effects of repeated testing, simulated malingering, and traumatic brain injury

**DOI:** 10.1371/journal.pone.0178148

**Published:** 2018-04-20

**Authors:** David L. Woods, John M. Wyma, Timothy J. Herron, E. William Yund, Bruce Reed

**Affiliations:** 1 Human Cognitive Neurophysiology Laboratory, VANCHCS, Martinez, California, United States of America; 2 UC Davis Department of Neurology, Sacramento, California, United States of America; 3 Center for Neurosciences, UC Davis, Davis, California, United States of America; 4 UC Davis Center for Mind and Brain, Davis, California, United States of America; 5 NeuroBehavioral Systems, Inc., Berkeley, California, United States of America; 6 Alzheimer’s Disease Center, Davis, California, United States of America; Instituto Cajal-CSIC, SPAIN

## Abstract

The Paced Auditory Serial Addition Test (PASAT) is widely used to evaluate processing speed and executive function in patients with multiple sclerosis, traumatic brain injury, and other neurological disorders. In the PASAT, subjects listen to sequences of digits while continuously reporting the sum of the last two digits presented. Four different stimulus onset asynchronies (SOAs) are usually tested, with difficulty increasing as SOAs are reduced. Ceiling effects are common at long SOAs, while the digit delivery rate often exceeds the subject’s processing capacity at short SOAs, causing some subjects to stop performing altogether. In addition, subjects may adopt an “alternate answer” strategy at short SOAs, which reduces the test’s demands on working-memory and processing speed. Consequently, studies have shown that the number of dyads (consecutive correct answers) is a more sensitive measure of PASAT performance than the overall number of correct sums. Here, we describe a 2.5-minute computerized test, the Dyad-Adaptive PASAT (DA-PASAT), where SOAs are adjusted with a 2:1 staircase, decreasing after each pair of correct responses and increasing after misses. Processing capacity is reflected in the minimum SOA (minSOA) achieved in 54 trials. Experiment 1 gathered normative data in two large populations: 1617 subjects in New Zealand ranging in age from 18 to 65 years, and 214 Californians ranging in age from 18 to 82 years. Minimum SOAs were influenced by age, education, and daily hours of computer-use. Minimum SOA z-scores, calculated after factoring out the influence of these factors, were virtually identical in the two control groups, as were response times (RTs) and dyad ratios (the proportion of hits occurring in dyads). Experiment 2 measured the test-retest reliability of the DA-PASAT in 44 young subjects who underwent three test sessions at weekly intervals. High intraclass correlation coefficients (ICCs) were found for minSOAs (0.87), response times (0.76), and dyad ratios (0.87). Performance improved across test sessions for all measures. Experiment 3 investigated the effects of simulated malingering in 50 subjects: 42% of simulated malingerers produced abnormal (p< 0.05) minSOA z-scores. Simulated malingerers with abnormal scores were distinguished with 87% sensitivity and 69% specificity from control subjects with abnormal scores by excessive differences between training performance and the actual test. Experiment 4 investigated patients with traumatic brain injury (TBI): patients with mild TBI performed within the normal range while patients with severe TBI showed deficits. The DA-PASAT reduces the time and stress of PASAT assessment while gathering sensitive measures of dyad processing that reveal the effects of aging, malingering, and traumatic brain injury on performance.

## General introduction

The Paced Auditory Serial Addition Test (PASAT) is a neuropsychological test in which digits are presented sequentially while subjects report the sum of each new digit and the immediately preceding digit. For example, if the digits “2”, “5”, and “3” are presented, the subject should respond with the sums “7” and 8”. The PASAT was developed by Gronwall and Wrightson (1974) to evaluate processing deficits in patients with traumatic brain injury (TBI) [1]. They presented 61 pseudo-random digits from audio tape at four different stimulus onset asynchronies (SOAs): 2.4, 2.0, 1.6, and 1.2 s. They found performance deficits in patients with mild TBI that usually resolved rapidly, a pattern that has since been seen in other studies [2], including studies of college football players with concussion [3]. In subsequent work, Gronwall and Wrightson (1981) found long-lasting deficits in patients who had suffered moderate and severe TBI [4].

There are two major variants of the original PASAT paradigm. Levin et al. (1982) [5] developed a 50-trial PASAT at four SOAs ranging from 3.0 to 1.6 s. Subsequently, Rao et al. (1991) [6] developed a version using SOAs of 3.0 s and 2.0 s that was incorporated into the Multiple Sclerosis (MS) Functional Composite score [7].

PASAT performance places demands on multiple cognitive abilities including attention, processing speed, working memory, and executive function [8]. Because of the multiple cognitive domains engaged, Gronwall found that weekly PASAT performance measures could guide occupational therapy decisions: TBI patients whose PASAT scores fell within one standard deviation of the normative mean were transitioned into return-to-work programs [9].

Nevertheless, the PASAT is currently used sparingly by most neuropsychologists [10] and has declined in popularity in recent years [11]. Four factors contribute to its sparse use: (1) The PASAT is stressful and aversive for many subjects, (2) Subjects often adopt an “alternate answer” strategy as SOAs are shortened, (3) There are inconsistencies in PASAT normative data that complicate test interpretation, and (4) Floor effects occur frequently at short SOAs, and ceiling effects occur frequently at long SOAs. Here, we review the current status of the PASAT and describe a new version, the Dyad-Adaptive PASAT, that reduces the stress of PASAT assessment, encourages a uniform processing strategy throughout the test, produces consistent results in different populations, and minimizes floor and ceiling effects.

Many subjects find the PASAT frustrating and stressful. As Tombaugh noted in his influential review [8], “Administration of the PASAT creates an undue amount of anxiety and frustration in participants which affects their performance on this and other neuropsychological tests, and may subsequently increase their reluctance to return for follow up testing.” Indeed, many subjects stop performing at the shorter, more demanding SOAs. For example, in the study of Vanderploeg et al. (2005) [12], testing was discontinued if the examiner felt that subject frustration would interfere with further neuropsychological assessments. Of the 3,057 control subjects who completed the PASAT at long SOAs, as SOAs decreased only 57% were tested at 1.2 s SOAs. Lopez-Gongora et al. (2015) [13] found that 5.3% of control subjects and 13.1% of patients with multiple sclerosis (MS) declined to perform the PASAT even at the longest (3.0 s) SOA, while Coo et al. (2005) [14] found that 16.1% of MS patients either declined to perform the PASAT or failed to complete testing at both 3.0 s and 2.0 s SOAs. Similarly, Strauss et al. (1994) [15] found that 21% of TBI patients were unable to perform the PASAT at shorter SOAs.

Interpreting the results of PASAT assessments is also complicated by inconsistencies in PASAT normative data. Table 1 shows digit-report accuracy in recent PASAT normative studies using the Gronwall, Levin, and Rao procedures. Significant differences in normative results are evident. For example, the accuracy of the subjects in Wiens et al. (1997) [16] at 2.4 s SOAs was nearly one standard deviation above the mean accuracy of the subjects of Diehr et al. (1998) [17], and nearly two standard deviations above the mean accuracy of the subjects of Brittain et al. (1991) [18] and Vanderploeg et al. (2005) [12]. Some of these differences may be due to demographic factors. For example, the subjects of Wiens et al. (1997) [16] were 9.2 years younger and had 1.3 more years of education than the subjects of Vanderploeg et al. (2005) [12]. However, a regression analysis relating age and education to PASAT performance [17] suggests that these demographic differences would account for only a small fraction of the observed differences in performance between the groups.

**Table 1:** PASAT normative studies.

Authors	N	Age	Edu	Trials	SOA (s)		
					3.0	2.4	2.0
**RAO PROCEDURE**							
Vanotti et al., 2016	296	44.0(16.3)	11.7 (4.4)	60	74.3 (17.9)		
Morrow et al., 2013	146	37.5(10.9)	14.3 (2.1)	60	79.3 (15.2)		
Drake et. al., 2009	100	44.6 (9.2)	14.6 (2.2)	60	80.0 (17.8)		
Cores et al., 2015	274	41.3 (10.0)	15.2 (2.1)	60	81.5 (16.0)		
Rao et al., 1991	100	46.0 (11.6)	13.3 (2.0)	60	80.3 (16.0)		61.8 (16.8)
Boringa et al. 2001	140	45.8	10.0	60	81.2 (17.8)		63.7 (18.3)
Ozakbas et al., 2016	385	36.0	9.0	60	72.0 (16.5)		
Amato et al., 2006	200	41.7 (9.6)	12.5 (3.7)	60	74.8 (20.1)		60.7 (20.1)
Sepulcre et al., 2006	152	45.2 (11.9)	11.8 (3.0)	60	76.6 (17.9)		61.6 (17.8)
Solari et. al, 2007	105	38.3 (9.3)	~12.0	60	72.3 (22.0)		55.7 (18.8)
**LEVIN PROCEDURE**							
Brittain et al., 1991	526	39.5	13.1	50		65.5	57.3
Diehr et al., 1998	566	37.9 (12.1)	14.2 (2.6)	50		76.3 (19.6)	63.3 (19.6)
Wiens et al., 1997	821	29.2 (6.0)	14.6	50		86.5 (11.4)	78.4 (14.1)
Roman et al., 1991	143	39.2	13.7	50		84.8 (11.6)	72.8 (16.2)
**GRONWALL PROCEDURE**							
Gronwall, 1977	80	~30.0	~13.5	60		77.0 (10.0)	67.0 (12.0)
Wingenfeld et al., 1999	168	21.0 (5.1)	14+	60		78.2 (15.7)	71.1 (16.5)
Vanderploeg et al., 2005	3057	38.4 (2.5)	13.3 (2.3)	60		64.3 (18.0)	
Stuss et al., 1987	60	39.6	14.5 (2.6)	60		76.1 (16.2)	67.6 (18.6)
**Mean**	**465**	**38.8**	**13.2**		**77.9**	**76.1**	**66.4**
**DA-PASAT**							
Exp 1a	1617	46.2 (11.8)	12.4 (3.4)	54	76.9 (8.1)		
Exp. 1b	214	40.6 (20.5)	14.8 (2.5)	54	76.8 (10.1)		

The percentage of correct trials is shown at SOAs of 3.0 s, 2.4 s, and 2.0 s for different test procedures, with the mean averaged across procedures. Standard deviations are shown in parentheses.

This implies that uncontrolled differences in PASAT administration and/or scoring can influence scores. For example, pre-test training likely influences performance. However, training procedures were not specified by Wiens et al. (1997) [16] or Vanderploeg et al. (2005) [12]. In other studies, training varies considerably. For example, Gronwall and Wrightson (1974) [1] explained the procedure with written examples and gave subjects ten practice trials using digits spoken by the examiner. Diehr et al. (1998) [17] gave subjects four sets of four practice trials, with additional practice provided if needed. Finally, in the MS Functional Composite PASAT [19], patients are given up to three training sets of ten practice trials, and proceed to testing if they provide two correct answers on any training set.

Scoring rules may also differ. For example, some administration manuals specify that correct responses must be given prior to the presentation of the next digit [8]. However, since correct-but-delayed responses occur frequently at short SOAs, such scoring rules will significantly underestimate accuracy [20]. Studies also differ in the instructions and encouragement given when subjects became confused during testing [8].

Finally, normative studies may be biased by subject self-selection, the exclusion of control subjects who fail to reach criterion performance during training, or by the exclusion of subjects who fail to complete testing at all SOAs. Although many studies suggest that a significant percentage of control subjects are unwilling or unable to complete the PASAT [12], most normative studies provide little information about the percentage of individuals who declined to participate, failed to complete training, or discontinued testing as SOAs were reduced [16, 17, 21].

### PASAT scoring

PASAT scoring commonly includes the number of correct responses at each SOA. Ceiling effects are frequently observed at longer SOAs. For example, the average performance of control subjects is only about one standard deviation below the maximum possible score at 3.0 s [6, 22–24] and 2.4 s [16, 21] SOAs. Ceiling effects can also be seen in clinical populations. For example, Sonder et al. (2013) [25] found that a high percentage of MS patients had perfect or near-perfect scores at 3.0 s SOAs, even in the first test session. Since PASAT scores improve with repeated testing (see Experiment 2 below), such ceiling levels limit sensitivity to performance changes over time.

To avoid ceiling effects, investigators often report composite scores, the total number of correct responses summed across the SOAs tested [26]. However, similar composite scores may reflect different processing capabilities and strategies as a function of the SOA. For example, a subject with good arithmetic ability but long response times (RTs) may perform well at SOAs of 2.4 and 2.0 s, but collapse at SOAs of 1.6 and 1.2 s., while other subjects may have worse performance at long SOAs but adopt an “alternate answer strategy” (reporting the sum of every other digit pair) at short SOAs to generate similar composite scores.

### Patterns of responding

In his review, Tombaugh (2006) [8] noted that some individuals “adopt an ‘alternate answer’ strategy of adding two numbers, skipping one, adding two numbers, skipping one, which makes the PASAT (particularly at short SOAs) easier and less sensitive to cognitive impairments.” For example, consider the digit string d1, d2, d3, d4, d5, d6, d7, and d8 presented at SOAs of 1.2 s. A subject adopting the alternate answer strategy would report the sums d1+d2, d3+d4, d5+d6 and d7+d8, skipping the intervening responses. Thus, sums would be reported at 2.4 s intervals rather than the 1.2 s intervals imposed by the test. Moreover, the demands on working memory are reduced because the subjects do not need to retain digits from one sum to the next. An alternate-answer strategy results in a maximum accuracy of 50%, which exceeds the accuracy observed at 1.2 s SOAs in the majority of normative studies reviewed by Tombaugh [8]. Tombaugh went on to speculate that the “alternate answer strategy” may be responsible for reduced PASAT sensitivity to cognitive deficits at short SOAs. Indeed, some investigators have suggested restarting the test if the subject appears to adopt an alternate answer strategy [17].

The use of a dyad score, the number of correct answers immediately preceded by a correct answer, has been proposed to avoid the confounding influence of the alternative-answer strategy. Research shows that the dyad score in MS patients is more closely correlated with white matter lesions [27] and the MS clinical course [14] than the total-correct score. The dyad ratio, the ratio of hits in dyads to total hits, has also proven clinically useful [28]. For example, a subject who reports the sums d1+d2, d2+d3*, d3+d4*, and d7+d8, would produce four correct sums including two correct dyads (starred), resulting in a dyad ratio of 50%.

### The adjusting PASAT

Royan et al. (2004) [29] developed the 200-trial adjusting PASAT using initial SOAs of 2.4 s that decreased by 20 ms following each hit, and increased by 20 ms following each miss. Answers were entered by the examiner using a numeric keypad and SOA adjustments were made automatically by computer. Minimum SOAs (minSOAs) averaged 1.56 s in young college students, and were similar whether the subjects were tested with digits one through nine or digits one through five. Response times, estimated from the latency of the examiners’ number-key entry, averaged 1.83 s.

A subsequent study of the adjusting PASAT showed high test-retest correlations for minSOAs (range 0.74 to 0.95) and significant learning effects with repeated testing [30]. Minimum SOAs on the adjusting PASAT also differed significantly between control subjects and patients with severe TBI [31], although 10.1% of the mixed population of TBI patients and controls were unable or unwilling to complete the 200-trial test. Test sensitivity was similar if only 100 trials were analyzed, and the authors speculated that further reductions in the number of trials could have been achieved without loss of sensitivity if larger SOA adjustments had been used [31].

### The DA-PASAT

Here, we introduce the Dyad-Adaptive PASAT (DA-PASAT). The DA-PASAT adjusts SOAs using a standard 2:1 staircase; i.e., SOAs are reduced following two successive, independent hits and increased following a single miss. Thus, for sequences with flawless performance, SOA reductions would occur after every other dyad. For example, in the sequence h-**H**-H-**H**, where h is a hit and H is a dyad hit, there would be two SOA reductions (shown in bold font) rather than three reductions, as would have occurred had SOAs been adjusted following each correct dyad.

In a 2:1 staircase, performance should stabilize at SOAs where subjects are 70% correct [32]. An examination of the results in Table 1 shows that the mean accuracy in the standard PASAT is generally above 70% at 2.4 s SOAs, but below 70% at SOAs of 2.0 s. Hence, minSOA values in the DA-PASAT would be expected to fall between 2.0 and 2.4 s. In addition, rather than using fixed SOA adjustments, SOA adjustments in the DA-PASAT are proportional to the SOA, making it possible to significantly shorten the test.

We describe four experiments with the DA-PASAT. In Experiment 1, two normative populations were used to characterize the influences of demographic factors (e.g., age, education, etc.) on DA-PASAT performance. Large populations were studied in different laboratories by different experimenters. As noted above, significant differences are often seen in PASAT norms obtained in different laboratories (see Table 1). We anticipated that the DA-PASAT’s improved procedural and scoring control would reduce between-laboratory differences. In Experiment 2, a group of 44 young subjects underwent three test sessions at weekly intervals to analyze DA-PASAT test-retest reliability and learning effects. In Experiment 3, we analyzed the effects of simulated malingering on DA-PASAT performance. Finally, in Experiment 4, we investigated DA-PASAT performance in 27 patients who had suffered mild or severe TBI.

## Experiment 1. Performance norms on the DA-PASAT

In Experiment 1, we analyzed the influence of four demographic variables on DA-PASAT performance: (1) ***Age***. Previous studies have shown that age has a significant influence on PASAT performance, particularly as SOAs are reduced [17, 18, 21, 33–35]. (2) ***Education***. Previous studies have observed highly significant correlations between education and PASAT scores [17, 23, 24, 34–38]. (3) ***Computer-use***. The number of daily hours of computer-use correlates with performance on computerized tests of processing speed [39–42] and memory [43], including significant correlations (independent of education) with scores on verbal fluency [44] and digit span [45] tests that do not require subjects to use a mouse. Computer users are also likely to have superior mathematical abilities, which are known to contribute to improved PASAT scores [46–48]. (4). ***Sex***. Although significant sex differences have been reported in some studies [49], most previous studies have found no significant sex differences in performance [16, 21, 34, 36–38]. Therefore, we anticipated significant influences of age, education, and computer-use on DA-PASAT performance, without significant differences between male and female subjects.

In Experiment 1a, we tested a community sample of 1617 subjects in Rotorua, New Zealand, who ranged in age from 18 to 65 years. These subjects had been recruited to investigate the consequences of H_2_S exposure from geothermal hot springs on cognition [50]. In Experiment 1b, we tested 214 volunteers recruited in Martinez, California, ranging in age from 18 to 82 years.

### Experiment 1: Methods

#### Ethics statement

Subjects in New Zealand gave informed written consent following procedures approved by both the Regional Ethics Committee in Rotorua and by the Institutional Review Board (IRB) for the UC Davis/VANCHCS CTSC, while subjects in California gave informed written consent following procedures approved by the IRB of the Veterans Affairs Northern California Health Care System (VANCHCS). The participants in California were paid for their participation.

#### Subjects, Experiment 1a

Of the 1617 subjects in Experiment 1a, all were between the ages of 18 and 65 (mean age = 46.2 years) and 40.0% were male. Subjects were selected using a stratification scheme designed to ensure a balanced distribution of residential H_2_S exposures. We excluded persons who were unable to speak and write English, were unable to visit the study clinic because of disability, blind people, and pregnant women. Subjects had an average United States equivalent of 12.4 years of education. Ethnically, the sample was primarily of European background (80.0%) and New Zealand Maori (15.6%). The remaining 4.4% represented a variety of ethnicities, none representing more than 1% of the sample.

Subjects indicated the daily hours of computer-use on a separate questionnaire containing an 8-point Likert scale, with the options of “1: Never; 2: Less than 1 hour per week; 3: Less than 1 hour per day; 4: 1–2 hours per day; 5: 2–3 hours per day; 6: 3–4 hours per day; 7: 4–6 hours per day; 8: More than 6 hours per day”. Subjects reported an average computer-use score of 4.77 (sd = 2.25), i.e., slightly less than 2 hours of computer use per day.

The PASAT was the fifth computerized test administered to subjects, following digit span [45], finger tapping [51], simple reaction time [40], and choice reaction time [42]. Additional neuropsychological tests were administered manually, including the digit symbol test of the Wechsler Adult Intelligence Scale [52], the Grooved Pegboard Test [53], the Hopkins Verbal Learning Test [54], and the National Adult Reading Test [55].

#### Apparatus and stimuli

Only digits one through five were used in the DA-PASAT. Previous studies have shown that performance on this restricted set of digits is not significantly different from performance on the full digit set in the adjusting PASAT [29], although the influence of arithmetic ability on PASAT performance is reduced when a restricted digit set is used [56].

Digits were selected randomly with the constraint that each digit occurred twice in each set of ten trials. Thus, unlike most PASAT tests, identical digits could occur successively, and similar sums could be correct on successive trials. Digits were presented binaurally through headphones at calibrated intensities of 80 dB SPL. The initial SOA was 3.5 s. The step size adjustment was 5% of the current SOA. Thus, the SOA step size was initially 175 ms, but was reduced to 100 ms as SOAs reached 2.0 s.

Most subjects were tested with 108 trials to evaluate the time needed to reach minSOA values. We found that most subjects rapidly reached minSOAs, and therefore limited the analysis reported here to the first 54 trials. Testing was performed in a quiet room using a standard Personal Computer (PC) controlled by Presentation^®^ software (Version 13.0, NeuroBehavioral Systems, Berkeley CA).

#### Software and data

An executable, open-source version of the DA-PASAT for Windows computers is available at http://www.ebire.org/hcnlab/. An Excel spreadsheet of the data is available at https://figshare.com/articles/Data_from_the_Dyad-Adaptive_PASAT/5021429.

#### Training

The paradigm was first explained to subjects with several examples, and subjects were warned that they might find the test demanding. Once the paradigm was explained, subjects were given 15 practice trials at a fixed SOA of 3.5 s. If training accuracy was below 50%, subjects were given additional blocks of 15 training trials (maximum 3 blocks). Accuracy during the final training block was recorded. Subjects were told prior to the test, “Don’t worry if you forget one of the digits or add them together incorrectly. Everybody makes mistakes on this task. If you get lost, just don’t say anything. Listen for the next two numbers and say their sum, and then continue saying the sum of the next numbers that you hear.”

Overall, 139 subjects (8.6% of the population) did not take the test, including 1.7% who refused to undergo training, 3.0% who stopped before completing 15 training trials, 3.9% who completed training but could not achieve accuracy scores above 50% correct, and 0.2% who performed above 50% correct during training but declined to continue the test. These subjects were assigned minSOAs of 3.5 s.

#### Subjects, Experiment 1b

In Experiment 1b, 214 subjects were recruited in Martinez, CA, from advertisements on Craigslist (sfbay.craigslist.org) and from pre-existing control populations. Subjects were required to meet the following inclusion criteria: (a) native fluency in the English language; (b) no current or prior history of psychiatric illness; (c) no current substance abuse; (d) no concurrent history of neurologic disease known to affect cognitive functioning; (e) on a stable dosage of any required medication; (f) auditory functioning sufficient to understanding normal conversational speech; and (g) visual acuity normal or corrected to 20/40 or better.

The subjects ranged in age from 18 to 82 years (mean age = 40.6 years), had an average education of 14.8 years, and were 57% male. Mean computer-use scores were 5.27 (1.97), i.e., subjects used computers for slightly more than 2 hours per day. Subject ethnicities were 64% Caucasian, 12% African American, 14% Asian, 10% Hispanic/Latino, 2% Hawaiian/Pacific Islander, 2% American Indian/Alaskan Native, and 4% other.

#### Apparatus and stimuli

The DA-PASAT was the twentieth test in the California Cognitive Assessment Battery (CCAB). Each CCAB test session included the following computerized tests and questionnaires: finger tapping [51, 57], simple reaction time [39, 41], Stroop, digit span forward and backward [45, 58], verbal list learning [59], verbal fluency [44], visuospatial span [43, 60], trail making [61], vocabulary, design fluency [62], the Wechsler Test of Adult Reading (WTAR), choice reaction time [39, 42], risk and loss avoidance, delay discounting, the DA-PASAT, the Cognitive Failures Questionnaire (CFQ) and the Posttraumatic Stress Disorder Checklist (PCL) [63], and a locally developed traumatic brain injury (TBI) questionnaire.

To assure that the subjects in Experiment 1b understood the task, we added self-paced practice trials prior to the training trials. In the self-paced practice trials, numbers were presented manually by the experimenter, with each new number presented only after the subject had produced the correct sum of the previous two numbers. After at least six correct self-paced trials, subjects underwent training at fixed 3.5 s SOAs, as described in Experiment 1a. Overall, 10.2% of subjects underwent more than one 15-trial training block. Average accuracy during the final 15 training trials was 82.8%. Overall, 7.0% of Experiment 1b subjects showed final training accuracy below 50% correct. However, unlike Experiment 1a, all subjects in Experiment 1b underwent DA-PASAT testing.

#### DA-PASAT scoring

Subjects’ responses in both experiments were scored by the examiner in real time by comparing the subject’s response with a monitor display showing the correct sum on that trial. In Experiment 1a, the experimenter pressed a response key if the sum was correct, while the absence of an experimenter response signaled either a miss or an incorrect answer. In Experiment 1b, the examiner responded separately for correct and incorrect sums, while the absence of a response signaled a miss.

SOAs were decreased by 5% following two hits and increased by 5% following each miss or incorrect answer using a standard 2:1 staircase. This adjustment, after each independent dyad, generally resulted in a rapid approach to the asymptotic SOA. For example, subjects producing twenty correct responses in a row at the beginning of the test would reduce SOAs by ten steps to reach 2.10 s by the twenty-first trial.

SOA adjustments were complicated by occasional late responses; i.e., trials where the response time (RT) exceeded the SOA. These occurred on 10.6% of the trials in Experiment 1a, and 9.4% of trials in Experiment 1b. Reaction times (RTs) on these trials exceeded the SOA by an average of 376 ms in Experiment 1a and 382 ms in Experiment 1b. Because the RT measure included both the time needed for the subject to articulate a response and the examiner’s reaction time, subjects had typically begun to articulate late responses before the end of the trial. However, since no response was recorded within the trial interval, the SOA would increase by 5% regardless of the correctness of the delayed response. Therefore, correspondingly larger SOA adjustments were made after the next trial following a delayed hit. For example, assuming an initial SOA of 2000 ms, in the sequence “hit (SOA = 2000 ms), dyad hit(SOA = 1900 ms), delayed dyad hit(SOA = 1995), dyad hit(SOA = 1796 ms),” the SOA would decrease by 5% ms after the second hit, increase by 5% after the third trial since no response was detected prior to the next trial, and decrease by 10% after the fourth trial when both the delayed hit on trial 3 and the correct response on trial 4 were detected.

#### Metrics

The primary measure of interest was the minSOA obtained during the first 54 trials. We also analyzed the dyad ratio, the ratio of hits in dyads to total hits. For example, in the trial sequence h-**H**-H-**H**-**m**-h-**H**-H-**m**-h-**H**-H-**m**, where h is a singlet hit, H is a dyad hit, m is a miss, and bold font indicates an SOA adjustment, SOAs would decrease by a net of one step (four down and three up), with seven dyad hits, three singlet hits, and three misses, producing a dyad ratio of 0.70. In addition, we measured the RT on each trial.

#### Statistical analysis

The results were analyzed with Analysis of Variance (ANOVA) using CLEAVE (http://www.ebire.org/hcnlab). Greenhouse-Geisser corrections of degrees of freedom were uniformly used in computing p values to correct for covariation among factors and interactions, with effect sizes reported as partial ω^2^. Pearson correlation analysis was also used with significance levels evaluated with Student’s t-tests. Multiple linear regression was used to evaluate the contribution of multiple demographic factors on performance and to factor out the contribution of demographic variables to create z-scores.

### Experiment 1: Results

#### Experiment 1a

**Fig 1:**
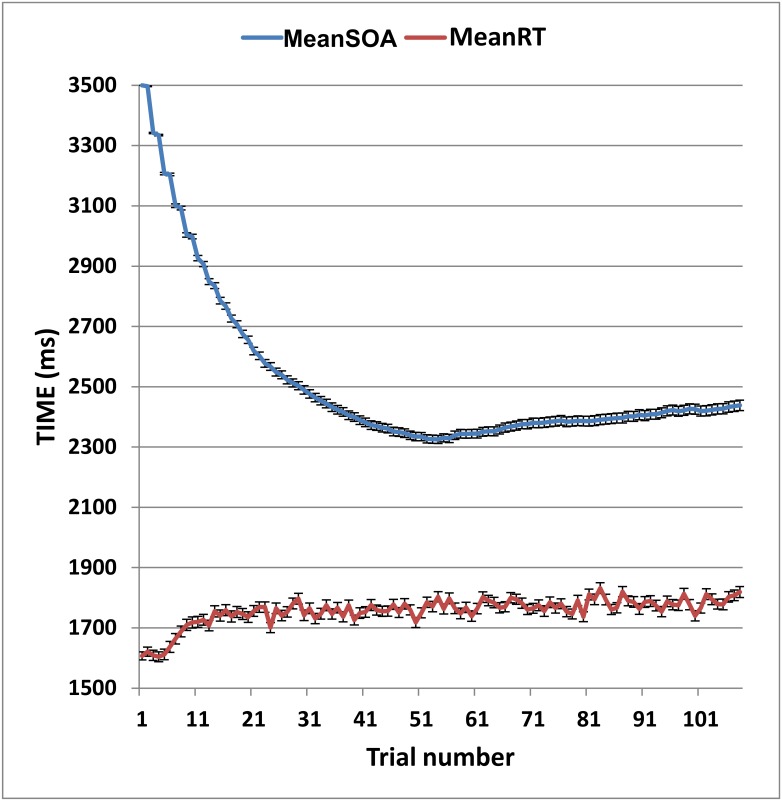
Mean SOAs and response times as a function of trial number for subjects in Experiment 1a. Error bars show standard error.

Fig 1 shows the mean SOAs for the subjects in Experiment 1a who received 108 trials. Mean SOAs declined sharply (by nearly 1.0 s) over the first 27 trials, and then continued to decline more gradually over the next 27 trials to reach a mean SOA of 2.33 s by trial 54. Testing terminated at trial 54 for 11.8% of subjects. For these subjects, the mean SOA was 3.70 s; i.e., most of these subjects could not maintain performance at 70% correct at 3.50 s SOAs, and SOAs increased as a result. For those subjects who received 108 trials, overall performance was well-characterized by the performance on the first 54 trials: the Pearson correlation of the minSOA by 54 trials and the minSOA over the entire test was 0.89, with an average difference of 0.15 s (0.19). On average, 54 trials required 2.5 minutes to administer.

**Fig 2:**
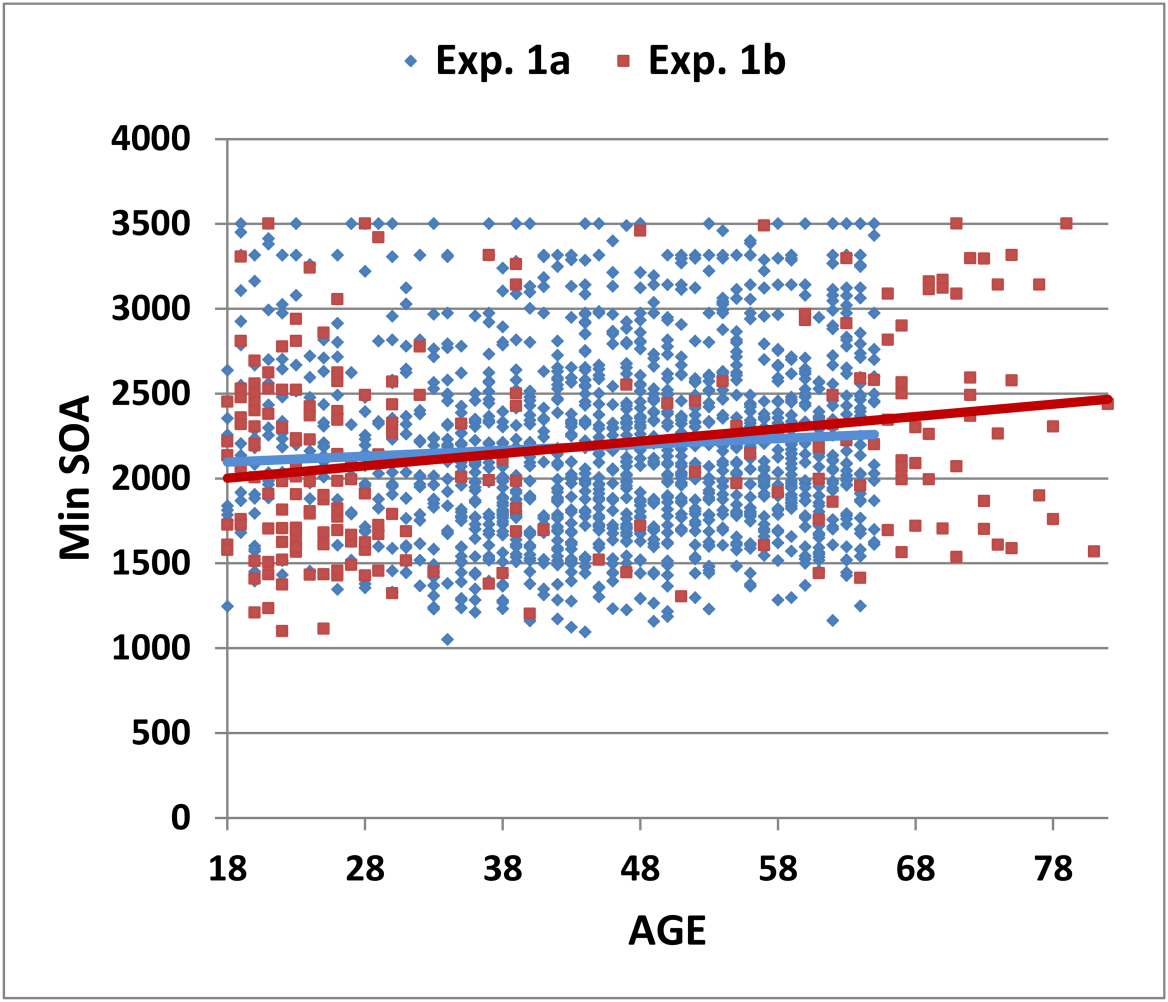
Minimum SOAs as a function of age. MinSOAs from the first 54 trials of subjects in Experiment 1a (blue diamonds) and Experiment 1b (red squares). Separate regression lines are shown for each experiment.

Fig 2 shows minSOAs over 54 trials as a function of age for the subjects in Experiment 1a (blue diamonds). Mean performance measures are shown in Table 2, and the correlation matrix for Experiment 1a is shown in Table 3 (upper right diagonals). Increased age was associated with increased minSOAs [r = 0.14, t(1615) = 5.68, p < 0.0001]. Both education [r = -0.23 t(1615) = -9.50, p < 0.0001] and computer-use [r = -0.25, t(1615) = = 10.40, p<0.0001] had significant influences on performance, with both correlations significantly stronger than that of age [z = -2.65, p < 0.01, and z = -3.25, p < 0.005, respectively]. Sex had a minimal, but statistically significant, influence on minSOAs [r = 0.08, t(1615) = 3.23, p < 0.002]; men had slightly lower minSOAs than women.

**Table 2:** Results from the different experiments.

	N	Age	Edu	C-use	MinSOA	Hits	DyRat	MinSOA z	RT
**Exp. 1a**	1617	46.2 (11.8)	12.4 (3.4)	4.8 (2.3)	2.17 (0.52)	41.6 (4.2)	0.78 (0.11)	0.00 (1.00)	1.80 (0.36)
**Exp. 1b**	214	40.6 (20.5)	14.8 (2.2)	5.3 (2.0)	2.14 (0.57)	41.4 (5.8)	0.79 (0.11)	0.05 (1.00)	1.86 (0.35)
**Exp. 2a**	44	26.2 (5.5)	15.4 (1.7)	5.9 (1.5)	2.04 (0.48)	42.9 (4.0)	0.83 (0.07)	0.04 (0.98)	1.69 (0.27)
**Exp. 2b**					1.77 (0.37)	44.9 (3.4)	0.86 (0.06)	-0.48 (0.79)	1.59 (0.26)
**Exp. 2c**					1.64 (0.37)	46.1 (5.0)	0.89 (0.05)	-0.73 (0.77)	1.48 (0.28)
**Exp. 3**	50	26.0 (5.4)	15.1 (1.9)	6.0 (1.6)	3.40 (0.64)	36.4 (8.4)	0.68 (0.18)	1.39 (1.15)	2.19 (0.54)
**mTBI**	23	32.9 (10.6)	13.4 (1.4)	5.0 (1.8)	2.30 (0.64)	40.2 (7.7)	0.78 (0.09)	0.24 (1.19)	1.87 (0.34)
**sTBI**	4	46.0 (9.0)	13.0 (1.2)	4.5 (2.7)	2.76 (0.89)	32.0 (14)	0.75 (0.16)	1.02 (1.44)	1.88 (0.18)

Edu = education. C-use = computer-use. MinSOA = minimal SOA. Hits = number of correct trials. DyRat = ratio of hits in dyad trials to total hits. MinSOA z = MinSOA z-score, with the influences of age, education, and computer-use factored out. RT = average response time in seconds. Except for z-scores, data are from subjects who completed at least 54 trials.

**Table 3:** Correlation matrices for Experiment 1a (above diagonal) and Experiment 1b (below diagonal).

	**Age**	**Edu**	**C-use**	**MinSOA**	**DyRat**	**MeanRT**
**Age**		-0.01	-0.09	0.14	-0.06	0.01
**Edu**	0.11		0.28	-0.23	0.13	-0.13
**C-use**	-0.20	0.39		-0.25	0.16	-0.14
**MinSOA**	0.23	-0.20	-0.28		-0.80	0.66
**DyRat**	-0.15	0.20	0.23	-0.80		-0.60
**RT**	0.11	-0.21	-0.23	0.73	-0.72	

See Table 2 for abbreviations. Given the number of observations, a significance level of p < 0.005 requires a correlation > |0.07| in Experiment 1a and |0.20| in Experiment 1b without Bonferroni correction. All data are from subjects who completed 54 trials.

**Fig 3:**
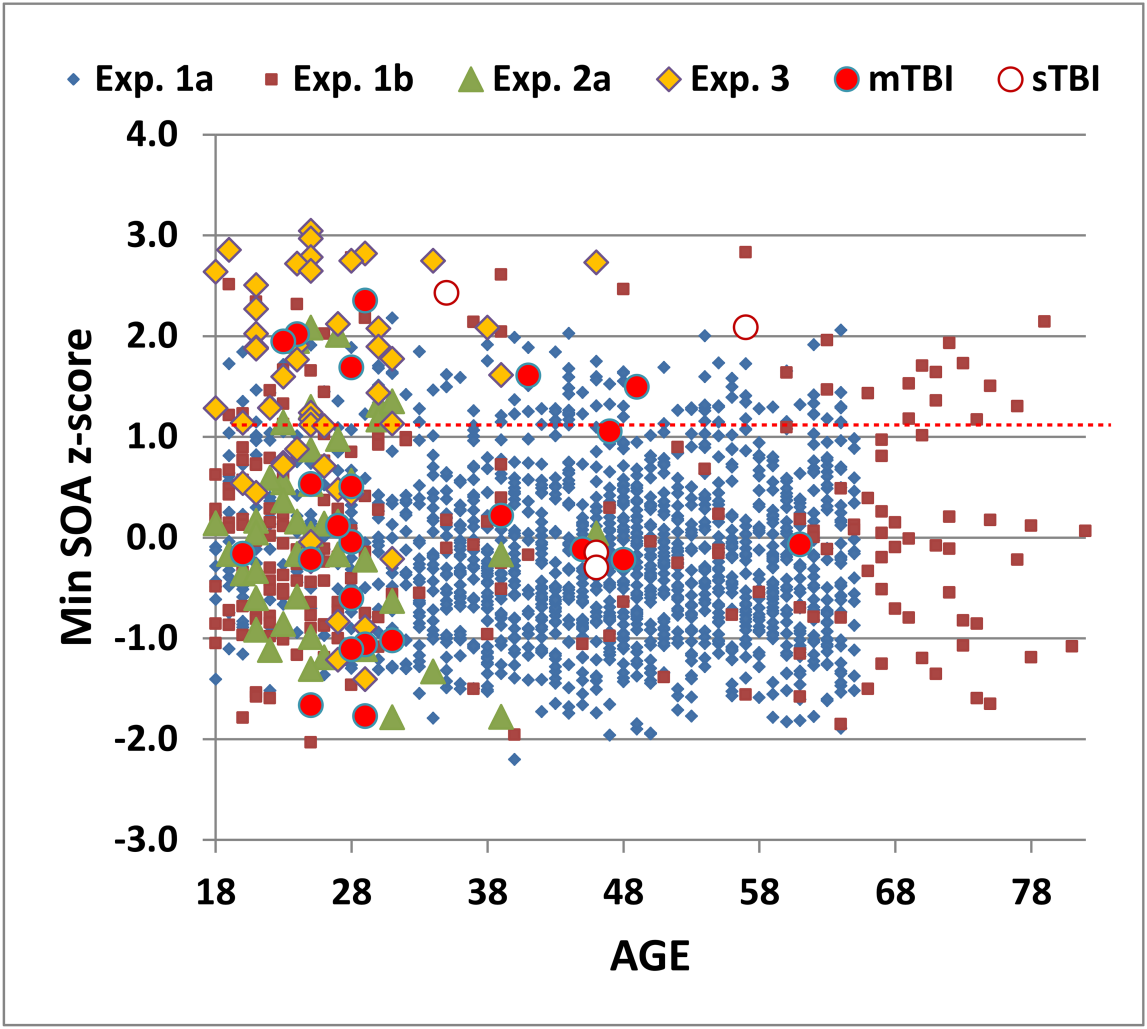
MinSOA z-scores. Data from 54 trials are shown for subjects of different ages in Experiment 1a (blue diamonds), Experiment 1b (red squares), Experiment 2a (green triangles), Experiment 3 (tan diamonds), patients with mild TBI (red circles), and patients with severe TBI (cross-hatched red circles). The dashed red-line shows the p < 0.05 abnormality threshold from Experiment 1a.

Multiple regression with Age, Education, and Computer-use as factors accounted for 10.5% of minSOA variance and revealed that each of the three factors had a significant effect [Age: t(1613) = 5.03, p < 0.0001; Education: t(1613) = -7.29, p < 0.0001; Computer-use: t(1613) = -7.67, p < 0.0001]. Subject z-scores were calculated after factoring out the influence of these factors using the regression equation minSOA = 2680.88 + 6.36*Age -33.56*Education -53.07*Computer-use. The minSOA z-scores for Experiment 1a subjects are shown in Fig 3 (blue diamonds).

Not surprisingly, dyad ratios declined as SOAs were reduced, averaging 0.82 in the first half of the 54-trial test, and 0.72 in the second half [F(1,1436) = 748.59, p <0.0001, ω^2^ = 0.15]. Dyad ratios are known to decline as SOAs are reduced in the standard PASAT [64], as would be expected; if hits followed a random distribution, the dyad score would be expected to decline more rapidly than the total correct score as SOAs are reduced [65]. For example, Fisk et al. (2001) [66] found dyad ratios of 0.72 at 2.4 s SOAs that declined to 0.55 at 2.0 s SOAs, and Walker et al. (2012) [67] found dyad ratios of 0.89 at 3.0 s SOAs that declined to 0.67 at 2.0 s SOAs.

**Fig 4:**
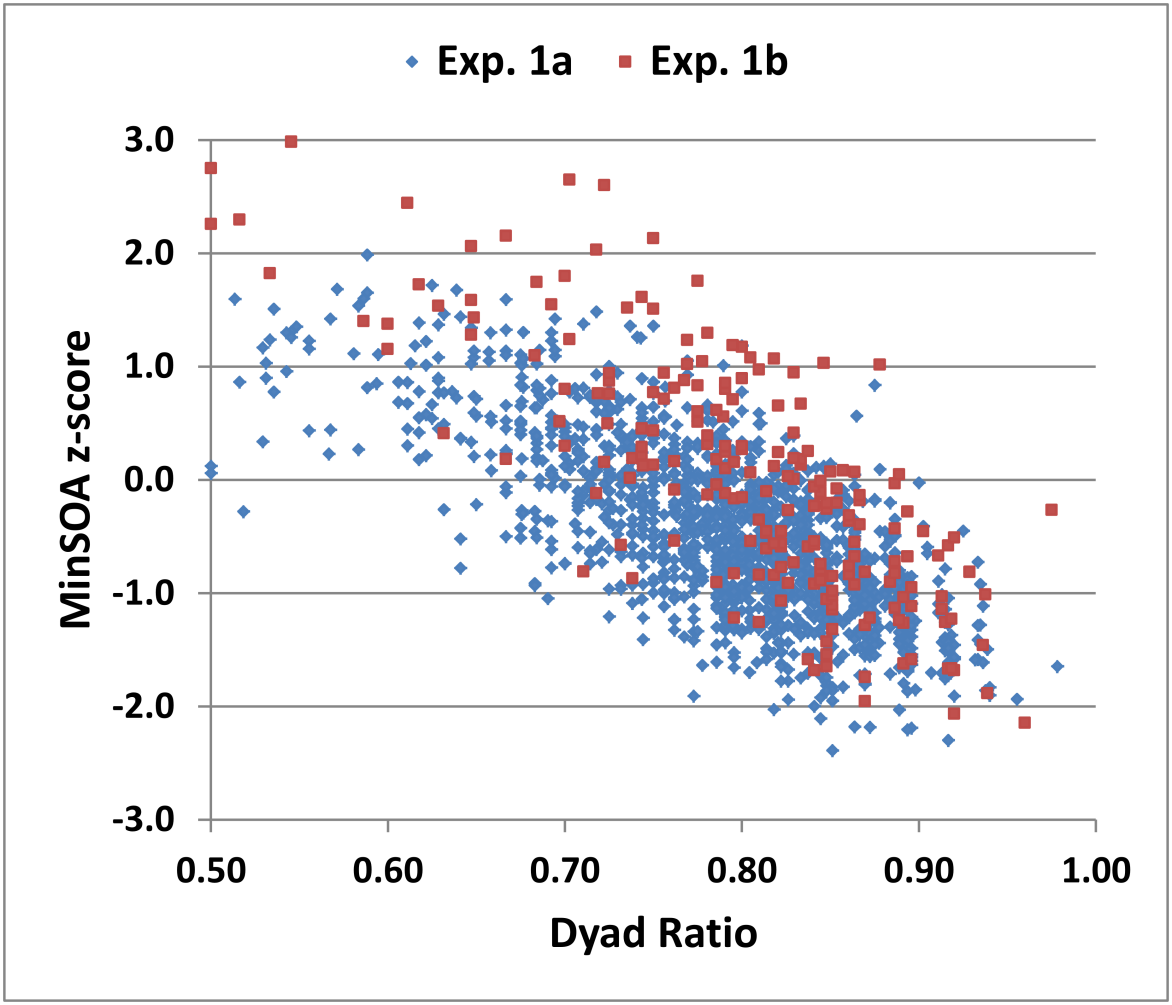
MinSOA z-score versus the dyad ratio (dyad-hits/total hits). Data are from Experiment 1a (blue diamonds) and Experiment 1b (red squares).

Fig 4 shows minSOA z-scores as a function of the dyad ratio in Experiment 1 (blue diamonds), revealing a strong negative correlation between the two measures [r = -0.65, t(1436) = -32.40, p < 0.0001]. Of the subjects with abnormal minSOA z-scores (i.e., in the highest 5% of minSOAs), 77% also had abnormal dyad ratios.

**Fig 5:**
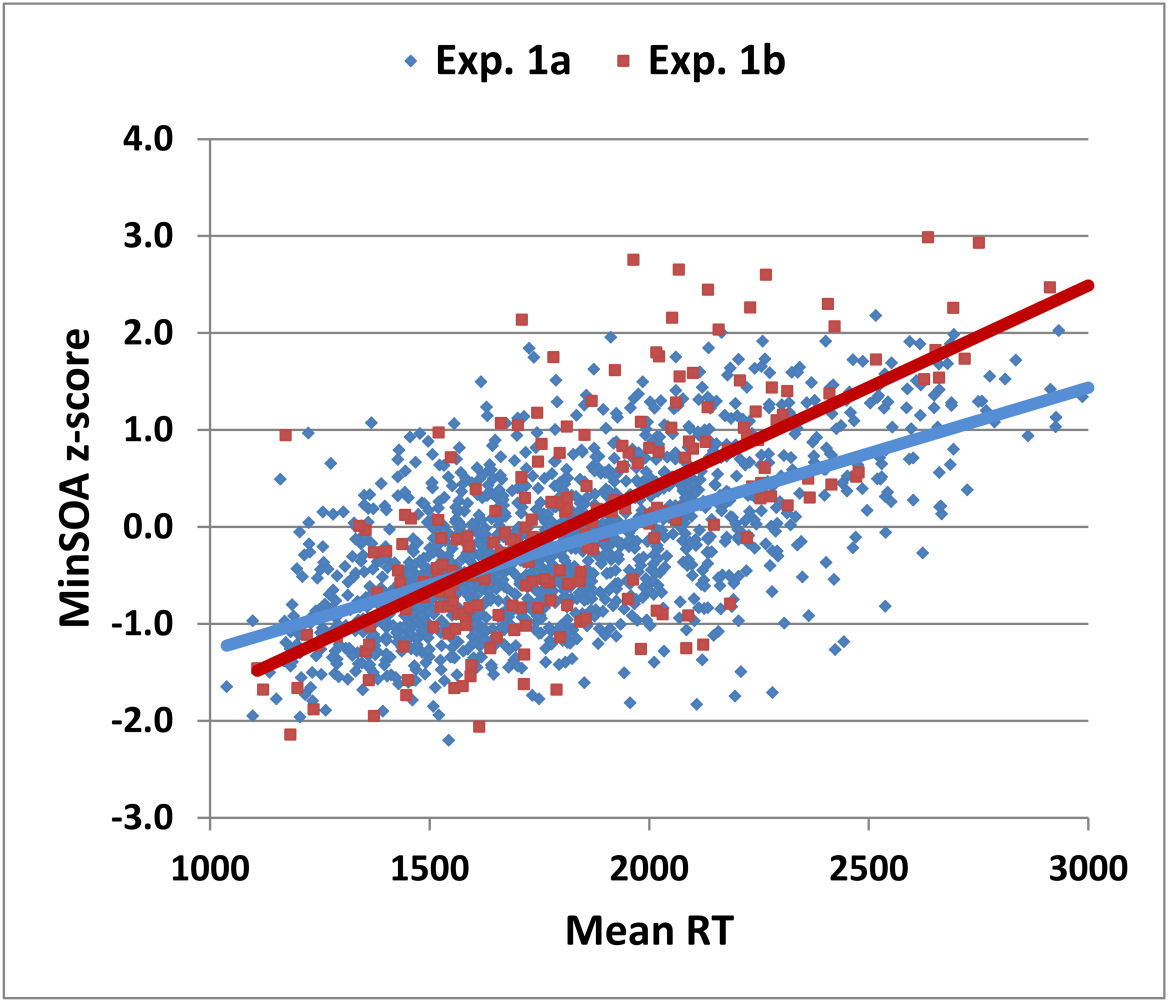
MinSOA z-score versus mean RTs. Data are from participants in Experiment 1a (blue diamonds) and Experiment 1b (red squares). Separate linear trend lines are shown for each experiment.

Response times in Experiment 1a averaged 1.80 s (0.36) and did not correlate significantly with age, but showed significant correlations (p<0.0001) with both education and computer-use (see Table 3). Multiple regression with Age, Education, and Computer-use as factors accounted for 3% of RT variance, with the age factor failing to reach significance [t(1433) = 0.86], but with significant influences seen for education [t(1433) = = 4.05, p < 0.0001] and computer-use [t(1433) = = 4.26, p < 0.0001]. As shown in Fig 5, there was also a strong correlation between mean RTs and minSOA z-scores [r = 0.57, t(1436) = 26.29, p < 0.0001]. Forty-three percent of subjects with abnormal minSOA z-scores had abnormal RTs.

#### Experiment 1b

In Experiment 1b, testing terminated at trial 54 for 15.4% of subjects. For the subjects who completed 108 trials, the Pearson correlation between the minSOA over 54 trials and the minSOA over 108 trials was r = 0.92. On average, 54 trials required 2.48 minutes to administer.

Summary results from Experiment 1b are included in Table 2. Subjects in Experiment 1b had lower mean age and higher levels of education and computer-use than the subjects in Experiment 1a, and minSOAs were slightly reduced (Fig 2, dark red squares). However, minSOA z-scores, calculated using the regression equation from Experiment 1a (Fig 3, dark red squares) were not significantly different from those in Experiment 1a [mean = 0.05, F(1,1829) = 0.93, NS].

The correlation matrix for Experiment 1b is included in Table 3 (below diagonal). As in Experiment 1a, minSOAs correlated significantly with Age, Education, and Computer-use (p <0.0001 for all correlations), and showed a strong negative correlation with the dyad ratio [r = -0.80, t(212) = -19.46, p < 0.0001]. However, unlike Experiment 1a, sex did not significantly influence minSOAs [r = 0.02, NS]. As in Experiment 1a, the three-factor regression (Age, Education, and Computer-use) accounted for more variance (13.0%) than a 2-factor regression with Age and Education (10.8%).

Dyad ratios in Experiment 1b did not differ from those in Experiment 1a [F(1,1649) = 2.93, p < 0.10] (see Fig 4), nor did RTs [F(1,1648) = 2.36, NS]. As in Experiment 1a, RTs correlated strongly with minSOA z-scores [r = -0.43, t(211) = -6.92, p < 0.0001] (see Fig 5), showed significant correlations with education and computer-use (p < 0.0001, see Table 3), and did not correlate significantly with age. Finally, as in previous reports [49, 68], we found that most errors in Experiment 1b were due to absent responses rather than incorrect sums: the ratio of incorrect sums to misses was 0.13 (0.31).

As in Experiment 1a, dyad ratios declined from the first half to the second half of the test [0.85 to 0.72, F(1,212) = 171.87, p< 0.0001, ω^2^ = 0.18]. Among subjects with abnormal minSOA z-scores (p <0.05), 44% had abnormal dyad ratios, and 38% had abnormally prolonged RTs.

### Experiment 1: Discussion

Minimum SOAs in the DA-PASAT reflected dyad-processing ability, with dyad ratios averaging 0.79. Most subjects began processing dyads at long SOAs and continued to process dyads as SOAs were reduced. Since the minSOAs over the first 54 trials correlated very strongly (0.89 to 0.92) with the minSOAs over all 108 trials, the DA-PASAT analysis was limited to 54 trials, which required approximately 2.5 minutes to administer.

#### Demographic factors influencing DA-PASAT performance

We found no significant differences in minSOA z-scores, RTs, or dyad ratios between the two control populations tested by different examiners in different laboratories. These results suggest that the improved procedural control provided by the DA-PASAT reduces the inter-laboratory differences that have plagued previous normative studies.

Demographic influences on the DA-PASAT were similar in the two control populations. We found significant age-related increases in minSOAs, consistent with previous studies that have shown a significant age influence on PASAT performance at SOAs below 2.0 s [17, 18, 21, 33–35]. In contrast, most studies have failed to find significant age-related differences at 3.0 s SOAs [23, 36, 37, 69], presumably due to the high incidence of ceiling effects.

We did not find a meaningful influence of sex on performance. In Experiment 1a, sex differences were statistically significant but minuscule, and sex differences were altogether absent in Experiment 1b. Although some previous studies have found significant sex differences of small magnitude [18, 35, 49], the majority of PASAT studies have reported comparable performance in men and women [16, 21, 34, 36–38]. As in previous studies, we found that performance improved with increased education. Although some previous studies have found small [16] or non-significant [18] effects of education, the majority have observed robust education influences [17, 23, 24, 34–38].

We also found that minSOAs correlated significantly with daily computer-use in both experiments. Moreover, the influence of computer-use remained significant after the effects of education had been factored out. The number of daily hours of computer-use may reflect subjects’ curiosity and intellectual engagement; it is correlated with performance both on computerized tests and on tests that do not require the subject to respond with or use the mouse, keyboard, or monitor, including the PASAT, verbal fluency [44], and digit span [45]. In addition, computer-use is associated with surrogate measures of IQ [44], and may be associated with mathematical ability, which is known to contribute to PASAT performance [46–48].

#### DA-PASAT measures of performance

In the DA-PASAT, the minSOA is determined by the number of misses versus the number of correct trial pairs, while the dyad ratio reflects the number of dyad hits relative to the total number of hits. The dyad ratio correlated negatively with minSOA z-scores. However, dissociations were sometimes evident between these two measures. For example, subjects in Fig 4 with minSOA z-scores around the mean had dyad ratios that ranged from 0.50 to 0.97, presumably reflecting interruptions in processing or variations in calculation speed.

Mean RTs in the DA-PASAT (1.89 s in Experiment 1a and 1.86 s in Experiment 1b) were similar to those obtained in the adjusting PASAT (1.83 s) [29]. Despite the strong correlation between RTs and minSOAs, there was some independence between minSOAs z-scores and RTs. For example, RTs varied from 1300 to more than 2500 ms among subjects with minSOA z-scores near the mean (Fig 5).

Thus, the combined analysis of dyad ratios and mean RTs can clarify the nature of processing deficits reflected in elevated minSOA z-scores. For example, 17 subjects in Experiment 1b had abnormal (p < 0.05) minSOA z-scores relative to Experiment 1a norms. Of these subjects, 44% had abnormal dyad ratios and 33% had abnormal RTs, with 28% showing both abnormalities. Forty four percent of the abnormal controls had dyad ratios and RTs that were both within the lower range of normal performance, suggesting that limitations in working memory and processing speed contributed conjointly to minSOA elevations.

#### Differences between the DA-PASAT and the adjusting PASAT

The DA-PASAT was designed to be less stressful than the adjusting PASAT [29–31]. Compared to the 1:1 staircase used in the adjusting PASAT, the 2:1 staircase in the DA-PASAT results in higher asymptotic accuracy (70% correct vs. 50% correct) and longer minSOAs (by about 0.70 s) [29, 31]. Finally, the DA-PASAT is of shorter duration (approximately 2.5 min vs. approximately 5.5 min) and begins at longer SOAs (3.5 s vs. 2.4 s). The more rapid approach to the minSOA asymptote is made possible by using adaptive SOA step-size adjustments (5% of the previous SOA) instead of the fixed 20-ms step size used in the adjusting PASAT.

## Experiment 2: Generalizability, test-retest reliability, and learning effects

Experiment 2 analyzed the results of repeated DA-PASAT testing in 44 young, well-educated subjects who underwent three test sessions at weekly intervals. We focused on three aspects of the results. First, to what extent would the results from Experiment 1 generalize to the first test session (2a) of Experiment 2? We anticipated that the subjects in Experiment 2a would have lower minSOAs than the subjects in Experiment 1 because they were younger, better educated, and used computers more frequently. However, we anticipated minimal differences in minSOA z-scores once these demographic influences had been factored out.

Second, we were interested in DA-PASAT test-retest reliability, a potential concern because of the short duration of the test. Previous studies have found high test-retest reliabilities for PASAT total-correct scores when averaged over SOAs (range 0.78 to 0.95) [8, 22, 70, 71] as well as high test-retest reliability (r = 0.94) for the adjusting PASAT [30].

Third, we were interested in the magnitude of learning effects. Previous studies have found significant increases in PASAT total-correct scores with repeated testing [25, 70, 72], and reductions in the minSOA with the adjusting PASAT [30].

### Experiment 2: Methods

#### Subjects

The test administration methods were identical to those described in Experiment 1b. Forty-four young volunteers (mean 26.2 years, range 18–46 years, 52% male) were recruited primarily from online advertisements on Craigslist. Subjects met the inclusion criteria of Experiment 1b and volunteered to participate in four weekly test sessions, including the three test sessions in Experiment 2. Mean age, education, and computer-use are shown in Table 2. Ethnically, 68% of the subjects were Caucasian, 11% Latino, 9% African American, 10% Asian, and 2% other.

#### Statistical analysis

The results were analyzed using the methods described in Experiment 1. In addition, intraclass correlation coefficients (ICCs) were analyzed with SPSS (version 25).

### Experiment 2: Results

Summary performance measures from Experiment 2 are included in Table 2. Average minSOAs in Experiment 2a were reduced with respect to Experiment 1a and 1b (see Fig 2). However, when adjusted for age, education, and computer-use, minSOA z-scores (0.04, see Fig 3) did not differ significantly from those of either Experiment 1a [F(1,1659) = 0.07] or Experiment 1b [F(1,256) = 0.03, NS]. As seen in Table 2, there were small differences in the dyad ratio and RT measures that were not corrected by demographic factors: in comparison with the subjects in Experiments 1a and 1b, subjects in Experiment 2a had increased dyad ratios [F(1,1479) = 11.14, p < 0.001, ω^2^ = 0.01; F(1,256) = 5.08, p< 0.03, ω^2^ = 0.02] and faster RTs [F(1,1479) = 4.72, p < 0.03, ω^2^ < 0.01; F(1,255) = 7.62, p< 0.01, ω^2^ = 0.03].

**Fig 6:**
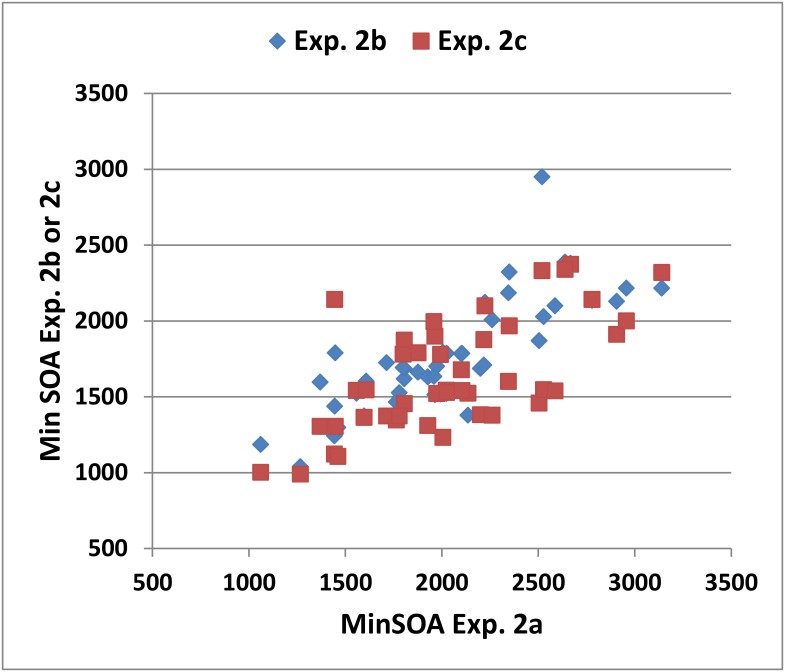
The reliability of minSOA measures in Experiment 2. MinSOAs from the 2^nd^ and 3^rd^ weekly test sessions (Experiment 2b and 2c) are plotted against minSOAs from the first test session (Experiment 2a).

Fig 6 shows the individual subjects’ minSOAs in Experiment 2a versus their minSOAs in Experiments 2b and 2c. High ICCs were found for minSOAs (0.87), dyad ratios (0.87), and mean RTs (0.76). In addition, we observed large learning effects: minSOA z-scores declined by 0.77 across the three test sessions [F(2,86) = 37.04, p < 0.0001, ω^2^ = 0.46], with significant differences between Session 1 and Session 2 (p< 0.0001) and between Session 2 and Session 3 (p < 0.0003). These changes were accompanied by increases in the dyad ratio [F(2,86) = 27.20, p < 0.0001, ω^2^ = 0.38] and reductions in RTs [F(2,86) = 12.61, p < 0.0001, ω^2^ = 0.21].

### Experiment 2: Discussion

The minSOA z-scores in Experiment 2a were well fit by the normative data in Experiment 1a and Experiment 1b. Small differences were seen in the dyad ratio and RTs, presumably because the subjects in Experiment 2 were younger, better educated, and used computers more frequently than the subjects in Experiment 1a or 1b. However, when demographic influences were factored out using the regression equation of Experiment 1a, no significant differences in minSOA z-scores were found when compared to either normative population.

Despite the test’s brevity, its test-retest correlations were similar to those obtained with the standard PASAT. For example, Lapshin et al. (2013) [73] found ICCs of 0.48 and 0.75 for a computerized, visual version of the PASAT using SOAs of 4.0 s and 2.0 s. Beglinger et al. (2005) [70] found test-retest correlations of 0.86 on the standard PASAT at 2.0 s SOAs, and 0.89 at 3.0 s SOAs, while Dyche and Johnson (1996) [71] found test-retest correlations of 0.90 for total correct responses among children of different ages. McCaffrey et al. (2001) [74] found correlations ranging from 0.90 to 0.96 for the total correct scores of control subjects tested at four different SOAs on five occasions over two years.

Large learning effects in the DA-PASAT were seen for minSOAs, the dyad ratio, and RTs, and were similar to the improvements observed when subjects undergo repeated testing with the standard PASAT [8, 25, 70, 72, 74] and the adjusting PASAT [30].

## Experiment 3: The effects of simulated malingering

The PASAT was originally developed to assess functional recovery in patients with traumatic brain injury (TBI) [1]. However, a significant percentage of TBI patients with litigation and pension claims show evidence of malingering on performance-validity tests (PVTs) [75, 76]. Only two studies have evaluated the effects of malingering on PASAT performance. Strauss et al. (1994) [15] examined PASAT performance in a handful of control subjects performing with full effort, a small control group instructed to simulate the effects of TBI, and 28 patients with mild to moderate TBI. They found that malingerers scored significantly below control subjects at 2.0 s SOAs, with differences reduced at 1.6 s SOAs, while the scores of TBI patients were intermediate between those of the control and simulated malingering groups. Proto et al. (2014) [77] compared PASAT performance among 178 veterans of the Iraq war who had suffered one or more mild TBIs (mTBI) and had been given six different PVTs. They found that PASAT scores were reduced at 2.4 and 2.0 s SOAs in patients with a greater number of PVT failures.

### Experiment 3: Methods

#### Materials and procedures

The 50 subjects in Experiment 3 included 40 subjects from Experiment 2 and ten subjects from Experiment 1b who agreed to participate in a simulated malingering test. Subjects had a mean age of 26.0 years and 55% were male. Their levels of education and computer-use are included alongside performance measures in Table 2.

The methods and procedures were identical to those of Experiments 1b, but subjects were given additional written instructions to feign the symptoms of a patient with mild TBI during a test session that included all CCAB tests the following week. The instructions were as follows: “Listed below you’ll find some of the symptoms common after minor head injuries. Please study the list below and develop a plan to fake some of the impairments typical of head injury when you take the test. Do your best to make your deficit look realistic. If you make too many obvious mistakes, we’ll know you’re faking! Symptom list: Difficulty concentrating for long periods of time, easily distracted by unimportant things, headaches and fatigue (feeling “mentally exhausted”), trouble coming up with the right word, poor memory, difficulty performing complicated tasks, easily tired, repeating things several times without realizing it, slow reaction times, trouble focusing on two things at once.”

#### A malingering index

An examination of the results from control subjects in Experiments 1a and 1b showed that most subjects with abnormal minSOA z-scores required multiple sets of training trials and continued to show low accuracy during the final training set. We hypothesized that simulated malingerers might show normal, or near-normal performance during training while manifesting impairments during testing.

#### Statistical analysis

The results were analyzed using Analysis of Variance (ANOVA) between groups to compare the results with those of the normative controls in Experiment 1a and 1b, and within-groups to compare performance during simulated malingering and full-effort conditions. Other procedures were identical to those of Experiment 1.

### Experiment 3: Results

The minSOA z-scores (mean 1.39) of the simulated malingerers were significantly elevated with respect to the subjects in Experiment 1a [F(1,1665) = 97.05, p < 0.0001, ω^2^ = 0.1] and Experiment 1b [F(1,251) = 60.40, p < 0.001 ω^2^ = 0.19], and with respect to the performance of the same subjects in their first full-effort test session [F(1,49) = 36.68, p < 0.0001, ω^2^ = 0.42]. Further analysis also showed corresponding reductions in dyad ratios and increases in mean RTs (p< 0.0001 for all comparisons).

Overall, 42% of malingering subjects showed minSOA z-scores in the abnormal (p < 0.05) range. Abnormalities in simulated malingerers tended to be greater than those of control subjects with abnormal scores (see Fig 3). However, as noted by Tombaugh (2006) [8], simple z-score cutoffs provided only moderate sensitivity and specificity in discriminating malingerers with abnormal minSOA z-scores (n = 23) from the abnormal controls in Experiment 1b. For example, 52% of simulated malingerers with abnormal scores had z-scores above 2.5 versus 25% of abnormal controls (52% sensitivity, 75% specificity).

We therefore analyzed performance consistency across training and test conditions. Control subjects with abnormal scores showed poor performance during training, with an average accuracy of 60% in the final set of training trials. In contrast, the performance of simulated malingerers during training (81% correct) was similar to that of the normal control population (87% correct). Using the bottom quintile of training accuracy as a cutoff, 87% of malingerers with abnormal z-scores showed training performance within the normal range seen in Experiment 1a, versus 31% of the controls with abnormal z-scores (i.e., 87% sensitivity and 69% specificity).

### Experiment 3: Discussion

Although we found a high incidence of performance deficits among simulated malingerers, z-score cutoffs were relatively ineffective at distinguishing between abnormal subjects in simulated malingering and control groups. This reflected the fact that the performance decrement due to malingering was relatively small, as has been noted by others [8, 15].

However, we found that training performance above the bottom quintile was rare in abnormal control subjects but relatively common in abnormal malingerers, showing a sensitivity of 87% and a specificity of 69%. This is consistent with previous results showing that simulated malingerers have difficulty adjusting the magnitude of performance deficits in different test conditions [39]. Adjusting performance deficits in training to match those during testing would be a relatively challenging task for malingering subjects without foreknowledge of the normal relationship between the two performance metrics.

#### Limitations

The magnitude of malingering deficits in minSOA z-scores was likely reduced by learning effects because the subjects in Experiment 3 were already familiar with the DA-PASAT, and their baseline performance had improved substantially as a result (see Experiment 2). Thus, insofar as malingering participants’ “abnormal” performance was relative to their current full-effort performance level, malingering deficits would have been reduced. In addition, test familiarity may have influenced their performance during training. Further studies of naïve simulated malingerers and patients suspected of malingering are needed to more fully evaluate the malingering sensitivity of the DA-PASAT and the reliability of training-score abnormalities in identifying malingerers.

## Experiment 4: The effects of traumatic brain injury on DA-PASAT performance

Gronwall and Wrightson (1974) [1] found that PASAT performance was impaired in patients with mild TBI (mTBI) when testing was performed in the acute phase, and other studies have since found similar PASAT deficits in acute mTBI [78–80]. However, the results of studies testing mTBI patients in the chronic phase (more than six months post-injury) are more ambiguous. For example, Leininger et al. (1990) [81] found a decrease of nearly one standard deviation in PASAT performance in a group of mTBI patients tested an average of seven months post-injury, and Cicerone and Azulay (2002) [82] found that mTBI patients with persistent cognitive symptoms showed PASAT abnormalities. In contrast, the large scale study of Vanderploeg et al. (2005) [12] found no significant differences in the performance of 3,057 control subjects and 254 mTBI patients tested in the chronic phase, although a larger percentage of mTBI patients refused to continue PASAT testing at SOAs below 2.4 s. Other studies have failed to find significant deficits in mTBI patients, with or without post-traumatic stress disorder (PTSD) [83]. Similarly, Tombaugh et al. (2006) [31] did not find significant differences between mTBI patients and controls on the adjusting PASAT. In contrast, patients with severe TBI (sTBI) generally show persistently impaired performance on the standard PASAT [1, 84–86] and the adjusting PASAT [31].

### Experiment 4: Methods

#### Subjects and procedures

The methods were identical to those of Experiment 1b. Twenty-seven Veterans with a history of TBI were recruited from the Veterans Affairs Northern California Health Care System patient population. The patients included 26 males and one female between the ages of 20 and 61 years (mean age = 35.5 years), with an average education of 13.6 years. All patients had suffered head injuries and a transient loss or alteration of consciousness, and had received diagnoses after extensive clinical evaluations. All patients were tested at least one year post-injury (see S1 Table). Twenty-three of the patients had suffered one or more combat-related incidents, with a loss of consciousness of less than 30 minutes, no hospitalization, and no evidence of brain lesions on clinical MRI scans. These patients were categorized as mTBI. The remaining four patients had suffered more severe accidents with hospitalization, coma duration exceeding eight hours, and post-traumatic amnesia exceeding 72 hours. These patients were categorized as sTBI. All patients were informed that the study was for research purposes only and that the results would not be included in their official medical records. Elevated scores (> 50) on the PTSD Checklist (PCL) were evident in the majority of the TBI sample, producing highly significant differences between TBI patients (mean PCL score = 53.9) and the control subjects (mean = 30.9) in Experiment 1b [F(1,209) = 71.11, p< 0.0001, ω^2^ = 0.25].

#### Statistical analysis

The results were analyzed with ANOVA, with separate comparisons of mTBI and sTBI groups with the control subjects in Experiment 1a and Experiment 1b.

### Experiment 4: Results

The minSOA z-scores are shown in Fig 3 for patients with mTBI (solid red circles) and sTBI (cross-hatched red circles). Mean performance measures for mTBI and sTBI patients are included in Table 2. The minSOA z-scores of mTBI patients (0.24, sd = 1.19) were not significantly different from z-scores obtained in Experiment 1a [F(1,1638) = 1.34, NS] or Experiment 1b [F(1,235) = 0.54, NS], nor were significant differences seen in dyad ratios [F(1,1458) = 0.91 and F(1,234) = 0.42] or RTs [F(1,1458) = 0.88 and F(1,235) = 0.15].

S1 Table includes minSOA z-scores from the individual TBI patients. Four mTBI patients had z-scores in the abnormal range defined in Experiment 1a (see Fig 3), an abnormality rate of 17.4%. All four abnormal mTBI patients showed relatively low accuracy during training. Relative to the normative data from Experiment 1a, the dyad ratios were in the abnormal range for two of the four abnormal mTBI patients, and near the abnormal range for the other two, while all four patients had normal RTs.

The minSOA z-scores of the sTBI patients (mean = 1.02, sd = 1.44) were significantly elevated with respect to minSOA z-scores in Experiment 1a [F(1,1619) = 4.31, p = 0.038], and showed a trend toward elevation with respect to Experiment 1b [F(1,216) = 3.24, p = 0.073]. Two of the four sTBI patients produced minSOA z-scores in the abnormal range. Neither showed excessive dissociations between training and test performance. Dyad ratios were abnormal for both patients, while RTs were within the normal range.

### Experiment 4: Discussion

The sensitivity of the DA-PASAT to TBI-related deficits appears to be similar to that of the traditional PASAT [12, 83] and adjusting PASAT [31]. As a group, mTBI patients tested in the chronic phase were not significantly different from control subjects. However, there was increased z-score dispersion, with 17.4% of z-scores falling into the abnormal range. None of the mTBI patients with abnormal scores showed signs of malingering, either during the PASAT or on other cognitive tests performed in the same test session [39, 41, 43, 61]. Our results are consistent with previous observations that a subgroup of mTBI patients may show persistent deficits in PASAT performance [1, 82].

Significant elevations in minSOA z-scores were observed in the small sTBI patient group, similar to the results of previous studies using the standard PASAT [84, 85] and the adjusting PASAT [31]. Patients with abnormal performance generally showed normal RTs and abnormal dyad ratios, consistent with deficits in working memory reported in this patient group [44, 58, 59].

#### Limitations

While the results of Experiment 4 provide preliminary evidence that the DA-PASAT is sensitive to deficits following TBI, larger patient studies are needed to more fully evaluate DA-PASAT sensitivity.

## General discussion

We performed four separate experiments to evaluate the DA-PASAT. Experiment 1 obtained normative data from two large populations. In contrast to the inter-laboratory differences in normative results gathered with standard PASAT paradigms (see Table 1), we found that minSOA z-scores, dyad ratios, and RTs were virtually identical in two control groups. The apparent improvement in the generalizability of normative data may reflect the improved procedural control in the DA-PASAT. Experiment 1 also confirmed previous studies showing that age and education significantly influence PASAT scores. In addition, another demographic variable, computer-use, was found to have a significant influence on performance that was independent of age and education. Thus, the precision of the regression functions used to calculate minSOA z-scores improved when computer-use was included along with age and education.

Experiment 2 investigated DA-PASAT test-retest reliability. The minSOA z-scores from the first test session of Experiment 2 were well fit by DA-PASAT norms from Experiments 1a and 1b. Repeated testing at weekly intervals showed high test-retest reliability for minSOAs and dyad ratios (both ICCs = 0.87), and only slightly lower reliability for RT measures (ICC = 0.76). Thus, the test-retest reliability of minSOA z-scores was similar to total-correct score reliability in previous PASAT studies [8]. Significant learning effects were also observed, as previously found following repeated testing with the standard PASAT [8] and adjusting PASAT [30].

Experiment 3 investigated the effects of simulated malingering. As in previous PASAT studies [8], malingering subjects showed significant performance impairments. However, the performance of simulated malingerers with abnormal scores showed considerable overlap with the performance of abnormal control subjects. Nevertheless, we found that simulated malingerers with abnormal scores could be discriminated from abnormal control subjects based on performance dissociations between training and test trials.

Finally, Experiment 4 demonstrated DA-PASAT sensitivity to TBI-related processing deficits that were similar to those seen in previous studies using the standard PASAT [8]. Most patients with mild TBI performed within the normal range, while patients with severe TBI showed abnormalities in minSOAs and dyad ratios. Reaction times remained within the normal range in TBI patients with abnormal minSOA z-scores, suggesting that their deficits primarily reflected impairments in working memory.

### Methodological improvements of the DA-PASAT

The DA-PASAT introduces five major improvements to PASAT assessment: (1) The DA-PASAT reduces subject stress and frustration by using SOAs adapted to each subject’s ability, measuring performance at high accuracy levels and minimizing test duration; (2) The DA-PASAT measures dyad-processing ability, the most sensitive metric of PASAT performance; (3) The DA-PASAT minimizes floor and ceiling effects by avoiding SOAs that are either too short or too long for each subject; (4) The DA-PASAT improves test efficiency by shortening test duration and reducing the potential errors associated with manual scoring; (5) Finally, the DA-PASAT collects multiple performance metrics that provide insight into PASAT performance: minSOA z-scores, dyad ratios, and mean RTs.

#### (1). Reduction of subject frustration and stress

Five design features of the DA-PASAT reduce subject stress and frustration compared to existing PASAT protocols. (1) Orientation and training is extensive to assure that subjects fully understand the task; (2) Testing starts at longer SOAs (3.5 s) than those used in other versions of the PASAT (3.0 s or 2.4 s); (3) Digits one to five are used rather than the digits one to nine, simplifying calculations and reducing the influence of mathematical ability [56]; (4) Subjects are tested at SOAs where they perform successfully (70% correct). In contrast, subjects in the standard PASAT are often tested at short SOAs beyond their processing capacity, resulting in accuracies below 50% [8]; (5) DA-PASAT testing requires approximately 2.5 minutes versus the eight to ten minutes required for standard PASAT protocols.

#### (2). Improved quantification of dyad processing

In the standard PASAT, dyad scores provide improved clinical sensitivity compared to total-correct scores [8, 14, 27, 28, 35, 56, 65, 66, 87]. However, dyad ratios may decline precipitously at short SOAs among control subjects who adopt an “alternate answer” strategy [8, 35, 66]. In contrast, the minSOA of the DA-PASAT reflects the subject’s dyad processing ability; subjects who adopt an alternate-answer strategy will show a systematic increase in SOAs.

#### (3). Reduction of floor and ceiling effects

The standard PASAT often shows floor effects at short SOAs because subjects become frustrated or cease performing altogether. In contrast, at longer SOAs (e.g., 3.0 and 2.4 s), ceiling effects are common at longer SOAs (e.g., 3.0 and 2.4 s), with many subjects achieving perfect or near-perfect scores Although the DA-PASAT begins at long SOAs (3.5 s), SOAs rapidly adapt to each subject’s capability so that floor and ceiling effects are avoided.

#### (4). Efficiency

The DA-PASAT requires about 2.5 minutes to administer, less than half of the time needed to administer the MS Functional Composite PASAT [6] and adjusting PASAT [29], and about a quarter of the time required to administer a 4-interval PASAT [5] [4] [17]. Moreover, the DA-PASAT is self-scoring, eliminating the time needed to tally responses and reducing potential scoring errors.

#### (5). Improved metrics

In the traditional PASAT, total-correct scores summed over SOAs are often used to measure overall performance. This can lead to ambiguities in interpretation, as similar combined scores can be produced by subjects with different strategies as a function of SOA. Test interpretation is further complicated by the discrepancies in the normative data collected in different laboratories. In contrast, the DA-PASAT minimizes ambiguities by measuring the maximal rate of successful dyad processing based on subject-specific SOA adjustments. Interpretation is based on the minSOA z-score after improved three-factor regression (age, education, and computer-use) using normative data that are consistent across different research laboratories, different experimenters, and different control populations.

Two additional measures help to more fully characterize DA-PASAT performance: dyad ratios and RTs. Dyad-ratios have been used with the standard PASAT [35, 65–67, 87], where they may depend on subject strategy. Some subjects attempt to maximize total correct scores at short SOAs by adopting an alternate-answer strategy, whereas others adhere to PASAT instructions despite a potential reduction in total-correct scores. In the DA-PASAT, minSOAs reflect the rate of successful dyad processing. The high dyad ratios observed with DA-PASAT testing (only 5% of normative subjects had dyad ratios below 0.62) confirm that the DA-PASAT is primarily assessing dyad-processing ability. Finally, the majority of control subjects with abnormal minSOA z-scores and all abnormal TBI patients had abnormal dyad ratios, consistent with deficits in working-memory capacity to retain digits across trials.

Response times also proved to be a useful supplementary measure. Tombaugh (2006) [8] noted that individuals who “speak slowly for cultural or geographic reasons” often show poor PASAT performance. Response times provide a measure of speech-production delays, and showed that speech delays had a relatively small influence in the control population: fewer than 10% of control subjects with abnormal minSOA z-scores had abnormal RTs but normal dyad ratios.

**Clinical applications**. The properties of the DA-PASAT make it a promising test for evaluating cognitive deficits in patients with TBI and other neurological disorders. Experiment 4 provides evidence that the DA-PASAT is sensitive to the processing deficits that follow severe TBI. Moreover, comparisons of performance during DA-PASAT training and the test appear to be sensitive to malingering, which can complicate the neuropsychological evaluation of patients with mild TBI [75]. However, larger studies are needed to more fully evaluate DA-PASAT sensitivity, including longitudinal studies to determine if DA-PASAT performance tracks recovery among patients with sports-related concussions and more severe head injuries. The evaluation of patients with MS would also benefit from the short duration of the DA-PASAT because it avoids the ceiling effects at 3.0 s SOAs that are often seen with the MS Functional Composite PASAT. In addition, RT measures might provide additional insight into the degree that speech delays contribute to the deficits seen in patients with MS and other disorders (e.g., aphasia).

## Conclusion

The Dyad-Adaptive PASAT is a 2.5 minute test that measures the ability of subjects to process dyads as SOAs are adaptively reduced based on performance. Because of the 2:1 staircase, SOAs in the DA-PASAT remain at or above the level where subjects can perform successfully, reducing the frustration and stress of the standard PASAT experienced by subjects tested at short SOAs. DA-PASAT interpretation is based on comparisons with normative data which show reduced inter-laboratory differences compared to the standard PASAT normative data sets. Finally, the DA-PASAT provides a core measure, the minSOA z-score, and further clarifies performance with supplementary dyad-ratio and reaction time measures.

## Supporting information

S1 Table Patient characteristics. Shaded cells show patients with severe TBI. PCL = scores on post-traumatic stress disorder checklist. See Table 2 for additional abbreviations.
(DOCX)
