# Supplementary material for: The Dyad-Adaptive Paced Auditory Serial Addition Test (DA-PASAT): Normative data and the effects of repeated testing, simulated malingering, and traumatic brain injury
Source: PLoS One. 2018 Apr 20;13(4):e0178148. doi: 10.1371/journal.pone.0178148 (PMC5909896; doi:10.1371/journal.pone.0178148)
Supplement: S1 Table — Shaded cells show patients with severe TBI. PCL = scores on post-traumatic stress disorder checklist. See Table 2 for additional abbreviations. (DOCX) [file pone.0178148.s001.docx]

| **S1 Table. Patient Characteristics** | | | | | | |
| --- | --- | --- | --- | --- | --- | --- |
|  | Age | Edu | C-use | PCL | minSOA-z | |
| PAT1 | 24 | 12 | 5 | 54 | | 2.02 |
| PAT2 | 28 | 12` | 4 | 66 | | 1.69 |
| PAT3 | 31 | 13 | 4 | 28 | | -1.02 |
| PAT4 | 41 | 14 | 4 | 45 | | 1.61 |
| PAT5 | 20 | 14 | 7 | 41 | | -0.16 |
| PAT6 | 25 | 15 | 7 | 87 | | 0.53 |
| PAT7 | 28 | 13 | 4 | 47 | | -0.04 |
| PAT8 | 25 | 12 | 6 | 57 | | -0.22 |
| PAT9 | 29 | 12 | 7 | 54 | | 2.35 |
| PAT10 | 47 | 14 | 5 | 52 | | 1.06 |
| PAT11 | 28 | 14 | 5 | 43 | | 0.51 |
| PAT12 | 29 | 13 | 4 | 27 | | -1.06 |
| PAT13 | 61 | 18 | 7 | 52 | | -0.07 |
| PAT14 | 27 | 15 | 4 | 72 | | 0.12 |
| PAT15 | 48 | 13 | 8 | 59 | | -0.22 |
| PAT16 | 49 | 12 | 1 | 47 | | 1.50 |
| PAT17 | 28 | 14 | 4 | 68 | | -0.60 |
| PAT18 | 39 | 13 | 2 | 64 | | 0.22 |
| PAT19 | 25 | 12 | 3 | 72 | | -1.66 |
| PAT20 | 45 | 14 | 8 | 60 | | -0.12 |
| PAT21 | 23 | 14 | 6 | 67 | | 1.95 |
| PAT22 | 29 | 14 | 6 | 41 | | -1.77 |
| PAT23 | 28 | 12 | 6 | 46 | | -1.11 |
| PAT24 | 35 | 12 | 5 | 59 | | 2.43 |
| PAT25 | 46 | 12 | 2 | 42 | | -0.15 |
| PAT26 | 57 | 14 | 3 | 56 | | 2.09 |
| PAT27 | 46 | 14 | 8 | 50 | | -0.30 |
